# Anthropogenic monoterpenes aggravating ozone pollution

**DOI:** 10.1093/nsr/nwac103

**Published:** 2022-05-31

**Authors:** Haichao Wang, Xuefei Ma, Zhaofeng Tan, Hongli Wang, Xiaorui Chen, Shiyi Chen, Yaqin Gao, Ying Liu, Yuhan Liu, Xinping Yang, Bin Yuan, Limin Zeng, Cheng Huang, Keding Lu, Yuanhang Zhang

**Affiliations:** State Key Joint Laboratory of Environmental Simulation and Pollution Control, State Environmental Protection Key Laboratory of Atmospheric Ozone Pollution Control, College of Environmental Sciences and Engineering, Peking University, Beijing 100871, China; School of Atmospheric Sciences, Sun Yat-sen University, Guangzhou 510275, China; Guangdong Provincial Observation and Research Station for Climate Environment and Air Quality Change in the Pearl River Estuary, Key Laboratory of Tropical Atmosphere–Ocean System, Ministry of Education, Southern Marine Science and Engineering Guangdong Laboratory (Zhuhai), Zhuhai 519082, China; State Key Joint Laboratory of Environmental Simulation and Pollution Control, State Environmental Protection Key Laboratory of Atmospheric Ozone Pollution Control, College of Environmental Sciences and Engineering, Peking University, Beijing 100871, China; Institute of Energy and Climate Research, IEK-8: Troposphere, Forschungszentrum Jülich GmbH, Jülich 52428, Germany; State Environmental Protection Key Laboratory of Formation and Prevention of the Urban Air Complex, Shanghai Academy of Environmental Sciences, Shanghai 200223, China; State Key Joint Laboratory of Environmental Simulation and Pollution Control, State Environmental Protection Key Laboratory of Atmospheric Ozone Pollution Control, College of Environmental Sciences and Engineering, Peking University, Beijing 100871, China; State Key Joint Laboratory of Environmental Simulation and Pollution Control, State Environmental Protection Key Laboratory of Atmospheric Ozone Pollution Control, College of Environmental Sciences and Engineering, Peking University, Beijing 100871, China; State Environmental Protection Key Laboratory of Formation and Prevention of the Urban Air Complex, Shanghai Academy of Environmental Sciences, Shanghai 200223, China; State Key Joint Laboratory of Environmental Simulation and Pollution Control, State Environmental Protection Key Laboratory of Atmospheric Ozone Pollution Control, College of Environmental Sciences and Engineering, Peking University, Beijing 100871, China; State Key Joint Laboratory of Environmental Simulation and Pollution Control, State Environmental Protection Key Laboratory of Atmospheric Ozone Pollution Control, College of Environmental Sciences and Engineering, Peking University, Beijing 100871, China; State Key Joint Laboratory of Environmental Simulation and Pollution Control, State Environmental Protection Key Laboratory of Atmospheric Ozone Pollution Control, College of Environmental Sciences and Engineering, Peking University, Beijing 100871, China; Institute for Environmental and Climate Research, Jinan University, Guangzhou 511443, China; State Key Joint Laboratory of Environmental Simulation and Pollution Control, State Environmental Protection Key Laboratory of Atmospheric Ozone Pollution Control, College of Environmental Sciences and Engineering, Peking University, Beijing 100871, China; State Environmental Protection Key Laboratory of Formation and Prevention of the Urban Air Complex, Shanghai Academy of Environmental Sciences, Shanghai 200223, China; State Key Joint Laboratory of Environmental Simulation and Pollution Control, State Environmental Protection Key Laboratory of Atmospheric Ozone Pollution Control, College of Environmental Sciences and Engineering, Peking University, Beijing 100871, China; State Key Joint Laboratory of Environmental Simulation and Pollution Control, State Environmental Protection Key Laboratory of Atmospheric Ozone Pollution Control, College of Environmental Sciences and Engineering, Peking University, Beijing 100871, China

**Keywords:** monoterpenes, ozone pollution, biomass burning, anthropogenic emissions, radical chemistry

## Abstract

Monoterpenes have been known to have a critical influence on air quality and climate change through their impact on the formation of fine particles. Here we present field evidence that monoterpene oxidations largely enhanced local ozone production in a regional site in eastern China. The observed monoterpene was most likely from biomass burning rather than biogenic emissions, as indicated by the high correlation with CO at night-time, and the observed ratio of these two species was consistent with previously determined values from biomass burning experiments. Fast monoterpene oxidations were determined experimentally based on direct radical measurements, leading to a daily ozone enhancement of 4–18 parts per billion by volume (ppb), which was 6%–16% of the total ozone production, depending on the speciation of monoterpenes. It demonstrates that the previously overlooked anthropogenic monoterpenes make an important contribution to O_3_ production in eastern China. The role could possibly be important at similar locations across China and other parts of the world that are characterized by massive emissions, especially where there are high NO*_x_* levels. Our results highlight that anthropogenic monoterpenes should be taken into account when proceeding with the coordinated mitigation of O_3_ and particulate matter pollution.

## Teaser

The previously overlooked anthropogenic monoterpene emission effectively worsens ozone pollution in eastern China, accounting for 6-16% of the total ozone production.

## INTRODUCTION

Monoterpenes are the second largest group of biogenic volatile organic compounds (BVOCs) in the atmosphere [1,2]. Their degradation by atmospheric oxidants, including hydroxyl radical (OH), nitrate radical (NO_3_) and O_3_, ultimately generates a variety of secondary pollutants [3–5]. These oxidation processes first generate complex organic peroxy radicals (RO_2_), subsequently form highly oxidized molecules under low nitrogen oxide conditions, and significantly contribute to new particle formation and secondary organic aerosol production [6–9]. Until now, many laboratory and field studies have focused on the effect of monoterpene oxidation on new particle formation and growth of secondary aerosol [10–20] while little attention has been paid to its impact on ozone production.

It is well acknowledged that ozone is produced during the photochemical oxidation of VOCs through the recycling of RO*_x_* radicals (e.g. OH, HO_2_ and RO_2_ radicals) by nitrogen monoxide (NO). The importance of isoprene and dominant anthropogenic VOCs in ozone formation is generally addressed [21–23]. However, large uncertainties and gaps remain in our ability to accurately predict the response of ozone to its precursors owing to the complexity and non-linearity of the photochemistry. Monoterpene chemistry is overlooked in ozone formation, because of the limited biogenic monoterpenes emitted in urban regions during the day. Recent studies indicate that significant emissions of anthropogenic monoterpenes, like volatile chemical products in urban regions in the USA, can have a significant impact on regional air quality [24–26]. Therefore, direct field evidence for the relationship between O_3_ formation and monoterpene oxidations would greatly improve our understanding of photochemical O_3_ pollution.

Here, we report field observations of monoterpenes and their oxidation rates versus OH, NO_3_ and O_3_, along with O_3_, NO*_x_*, anthropogenic VOCs, isoprene, OH, N_2_O_5_ and other related parameters, at a regional site in eastern China from 27 May to 8 June 2018 (see Materials and Methods, and Text S1 in the supplementary materials online). The comprehensive data set enables us to address the sources, the fate and the atmospheric impacts of monoterpenes. We show a fast monoterpene oxidation rate in air masses influenced by both anthropogenic and biogenic emissions in the Yangtze River Delta metropolitan areas, and reveal the significant role anthropogenic monoterpene oxidation plays in photochemical O_3_ production.

## RESULTS AND DISCUSSION

### Experimentally determined fast oxidation of monoterpenes

**Figure 1.**
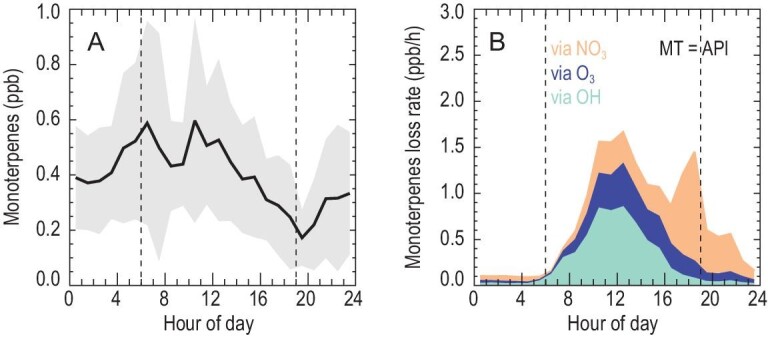
(A) Mean diurnal profile of monoterpenes and (B) the loss rates of monoterpenes observed at a regional site in summer in eastern China. The shadow in panel A represents the standard deviation of total monoterpenes. The colored areas in panel B show the monoterpene (identified as α-pinene, API) loss rates by different oxidants. The dotted lines in both panels denote sunrise and sunset time, respectively. MT is the abbreviation for monoterpenes.

Figure 1 shows the mean diurnal profiles of monoterpenes and the experimentally determined oxidation rates of monoterpenes through OH, O_3_ and NO_3_, respectively. Here, in the absence of further information regarding monoterpene speciation, all monoterpenes are assumed to be *α*-pinene, which is justified to some extent as *α*-pinene is the major monoterpene [27,28]. The uncertainty due to the simplification of monoterpenes is also tested by assuming that the observed monoterpenes are a much more reactive species, limonene. High monoterpene concentrations with an early morning peak and a near-noon peak are observed (Fig. 1A). The daytime (06:00–18:00) averaged concentration is 0.37 parts per billion by volume (ppb), which is comparable to the observed isoprene concentrations (0.34 ppb).

During this campaign, the hourly mean diurnal maxima of OH and O_3_ were 1.0 × 10^7^ cm^–3^ and 92.6 ppb, respectively (Figs S1 and S2 in the supplementary materials online), which are representative of the conditions in urban agglomeration regions in eastern China in warm seasons, and are generally higher than those reported elsewhere [29–31]. The daytime NO_3_ concentrations are experimentally derived from the steady-state calculation [32–34] (see Materials and Methods, and Text S1). The average daytime NO_3_ concentration is 1.6 parts per trillion by volume (ppt), which is also much higher than those previously reported in other regions [27,32,34]. The high daytime NO_3_ level is attributed to its high precursors (Fig. S3). The co-elevated daytime OH and monoterpene concentrations lead to a fast OH-induced monoterpene oxidation rate of 0.43 ppb/h, which accounts for 51.8% of monoterpene losses during the day. The monoterpene oxidation rate driven by NO_3_ reaches an average of 0.20 ppb/h in the daytime, which is equal to that caused by O_3_, and more than one order of magnitude higher than that reported in the southeast USA [35]. A similar fast daytime NO_3_-driven monoterpene oxidation rate was reported in a high NOx and O_3_ air mass on the Seoul tower [27]. This implies that NO_3_-induced monoterpene oxidation might have an impact on photochemistry.

Combined with the monoterpene oxidation by OH, O_3_ and NO_3_, we demonstrate an unprecedented fast monoterpene oxidation rate of 0.83 ppb/h on average in the daytime in this field observation (Fig. 1B), even exceeding the daytime isoprene oxidation rate of 0.75 ppb/h. Given that *α*-pinene is the least reactive monoterpene with respect to OH [36], the monoterpene oxidation rates, and therefore the estimated impact on ozone formation, represent a lower bound. When assuming that observed sum monoterpenes are limonene, a further high monoterpene oxidation rate would be derived (Fig. S4). It should be noted that the fast oxidation of monoterpenes is a result of simultaneous high concentrations of the monoterpenes and the oxidants, and thus it raises the question: what is the source that sustains such high monoterpene concentrations?

### Anthropogenic monoterpene emissions

**Figure 2.**
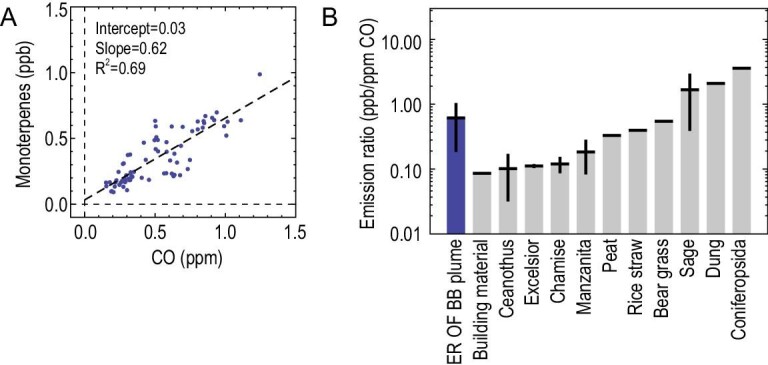
(A) The correlation of observed monoterpenes and CO during nighttime and (B) an intercomparison of a field-derived emission ratio of monoterpenes to CO, with the experimental results from biomass burning activities. The leftmost bar in panel B is the field-derived emission ratio and the others are obtained by laboratory experiments [40]. The error bar shows the standard deviation.

Several studies demonstrate that monoterpenes are not only from biogenic emissions but also from anthropogenic activities. Laboratory simulation experiments indicate that biomass burning of various kinds of crops can release high amounts of monoterpenes into the atmosphere. Since this campaign was conducted in a harvest season, biomass burning events occurred frequently during the campaign [37–39]. As shown in Fig. 2A, the observed monoterpenes show good correlation (R^2 ^= 0.69) with biomass-burning-emitted CO during night-time. The data set is restricted to night-time only, when oxidants (OH, O_3_) are at low levels, to avoid the effect of the fast loss of monoterpenes during the day and reflect the real emission ratio between monoterpenes and CO to some extent. The estimated emission ratio of monoterpenes to CO in the biomass burning plumes during this campaign is ∼0.7 ppb/ppm, which is comparable to those obtained from laboratory simulation experiments that involved the burning of different crops [40] (Fig. 2B). This indicates that biomass-burning-emitted monoterpenes contributed a large portion of the observed sum of monoterpenes during this campaign. Our results provide field evidence that biomass burning is a significant source of monoterpenes in eastern China.

### Monoterpene oxidation strongly enhances ozone formation

The rapid daytime oxidation of monoterpenes by OH, O_3_ and NO_3_ can efficiently trigger the production of peroxy radicals and significantly increase photochemical ozone production under elevated NO_x_ levels. In the meantime, the oxidation of monoterpenes by O_3_ can also provide an additional OH radical source [41], contributing ∼6% to the total OH primary source during the day (Fig. S5); it therefore plays a dual role in promoting ozone production by offering additional reactivity and a primary radical source. The net ozone production, P(O*_x_*), induced by monoterpene oxidation can be quantitatively determined by differentiating the P(O*_x_*), derived from two box model runs based on Regional Atmospheric Chemical Mechanism version 2 (RACM2), with and without constraint to monoterpenes observation (see Materials and Methods).

**Figure 3.**
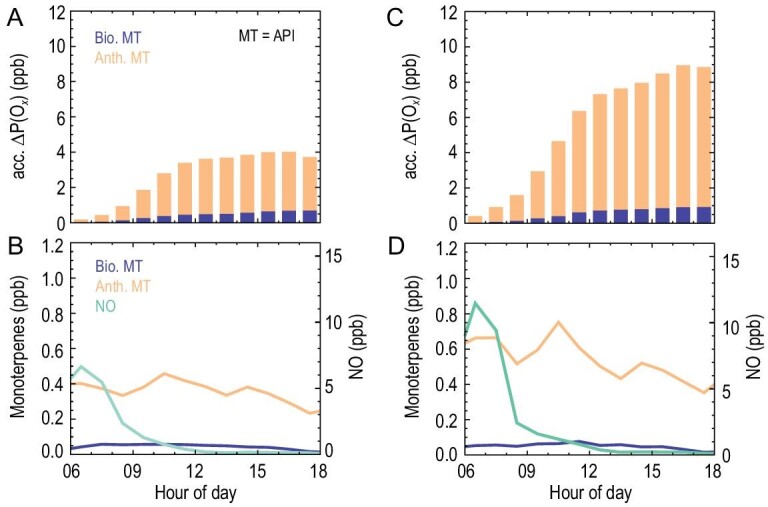
The enhancement of ozone production by monoterpene (MT) oxidation (identified as be α-pinene, API). (A, C) The ozone enhancements from monoterpene oxidation are denoted by daytime (06:00–18:00) accumulated net ozone production rates, and contributions from anthropogenic and biogenic monoterpene oxidation are separately shown. (B, D) The mean diurnal profiles of biogenic and anthropogenic monoterpenes, and nitrogen monoxide (NO). Panels A and B denote the mean diurnal profiles for the whole campaign. Panels C and D denote the mean diurnal profiles for the first 4 days (from 27 to 30 May).

As shown in Fig. 3A, the oxidation of monoterpenes is quantified to contribute an additional 4.0 ppb of ozone production per day on average (from 06:00 to 18:00) if we allocate observed monoterpenes to α-pinene, accounting for 6% of the daily integrated net ozone production. To underline the significance of anthropogenic activities on ozone production, we extract the biogenic emitted monoterpenes from the observed monoterpenes by applying a fixed emission ratio of biogenic emitted isoprene to monoterpenes. This ratio is assumed to be 4.65 in this study, the lower limit that the literature recommends (4.65∼12.14), which represents the largest contribution of monoterpenes from biogenic emissions [42–44]. The diurnal variations of monoterpenes that have originated from different sources (biogenic and anthropogenic) are shown in Fig. 3B. As a result, anthropogenic monoterpenes make up 89% of monoterpenes during daytime, with the peak appearing in the late morning, and they contribute 83% of the total enhancement of net ozone production by monoterpene oxidation. Notably, ozone production by monoterpene oxidation is mostly pronounced in the late morning when both monoterpenes and NO are relatively high. After midday, although monoterpene concentration remains, the observed NO concentration drops to below 0.20 ppb, which is insufficient to propagate the radical chain reaction effectively when the ozone production is NO*_x_*-limited, and thus limits the ozone production. In the meantime, ozone destruction through ozonolysis of monoterpenes is enhanced simultaneously due to the increased ozone concentration in the afternoon.

Furthermore, if we concentrate on the first 4 days, when biomass burning is observed to be more active (Fig. 3D), the ozone enhancement by monoterpene oxidation increases to 8.9 ppb during the day, accounting for 13% of the daily integrated net ozone production (Fig. 3C), 90% of which is attributed to anthropogenic monoterpene oxidation. If we assume that observed sum monoterpenes are limonene, the role of monoterpene oxidation on ozone production will be much more significant (Fig. S6), which leads to an additional 17.9 ppb and 33.8 ppb of ozone production per day (from 06:00 to 18:00) for the whole campaign and the first 4 days averaged, respectively. The cases for identifying monoterpenes as limonene are considered to be an upper limit to account for possible uncertainties with regard to monoterpene speciation in the measurements.

### Dependence on nitrogen oxides for ozone enhancement by monoterpenes

**Figure 4.**
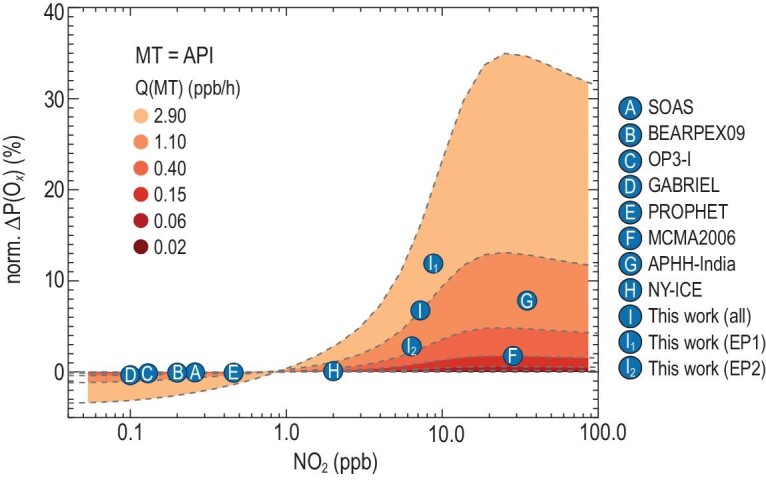
The dependence of calculated ozone enhancements from monoterpene chemistry on the concentration of nitrogen oxide. The ozone enhancements from monoterpene oxidations are denoted by the normalized increased-ozone production rates, norm. ΔP(O*_x_*) (see Materials and Methods). All monoterpenes are identified as be α-pinene (API). The letters on the circle points denote different measurement campaigns. A: SOAS, the Southern Oxidant and Aerosol Study, 2013 [45]. B: BEARPEX09, the Biosphere Effects on Aerosols and Photochemistry Experiment II, California [46]. C: OP3-I, Oxidant and Particle Photochemical Processes above a South-East Asian tropical rainforest: first intensive measurements [47]. D: GABRIEL, Guyanas Atmosphere-Biosphere Exchange and Radicals Intensive Experiment with the Learjet [48]. E: PROPHET, Program for Research on Oxidants: Photochemistry, Emissions and Transport [49]. F: MCMA2006, Exploratory field measurements in the Mexico City Metropolitan Area, 2006 [50]. G: APHH-India, the Atmospheric Pollution and Human Health program in an Indian Megacity [51]. H: NY-ICE, the New York Investigation of Consumer Emissions [25]. I: this study. The averaged conditions are further divided into two parts (I1, from 27 to 30 May; I2, from 2 to 8 July).

The impacts of the daytime oxidation of monoterpenes on the local ozone production rate towards NO_x_ are further extensively investigated through sensitivity tests for varied NO_x_ conditions. The model is applied to different monoterpene emission rates estimated from previous field observations. The modeled ozone production rates, P(O*_x_*), for different sensitivity cases are normalized to the maximum P(O*_x_*) with zero monoterpene emission (max. P(O*_x_*)_MT = 0_). The differences between the normalized P(O*_x_*) for certain monoterpene emission rates and that for the zero monoterpene emission case are plotted against NO_2_ to show the relative enhancement due to additional monoterpene emission (see Materials and Methods, Supplementary Data). This normalization works as a quantitative method to represent the ozone enhancement from monoterpene chemistry, and it allows us to synthesize the results from different campaigns. Figure 4 shows the impact of the monoterpene chemistry on the simulated ozone enhancements for varied NO*_x_* concentrations. A relatively small influence of monoterpene chemistry is deduced for the low NO_2_ regime (i.e. NO_2_ < 2 ppb) or even negative effects are found because monoterpenes efficiently consume both O_3_ and NO_3_. This influence on the ozone enhancement has the potential to be significant with NO_2_ > 3 ppb. The ozone enhancement increases with NO_2_ and reaches a maximum around 20 ppb of NO_2_, then slightly decreases with a higher NO_2_ level due to the competing radical loss processes that reduce the radical propagation chain length. In addition, the ozone enhancement due to monoterpene oxidation is proportional to the emission rate of the monoterpenes and the maximum enhancement also shifts towards a higher NO_2_ with larger monoterpene emission rates. For example, when monoterpene emission equals 1.1 ppb/h, the resultant maximum ozone enhancement is 13% around 20 ppb of NO_2_. This maximum ozone enhancement increased to 35% around 25 ppb of NO_2_ when the monoterpene emission rate was set as 2.9 ppb/h. The chemical condition for the averaged case of this campaign locates slightly left of the norm P(O*_x_*) peak. The slight difference between the two episodes (first 4 days and last 7 days) during the campaign is clearly shown in the NO_2_ dependence. Relatively higher NO*_x_* and monoterpenes coexisted during the first 4 days. In this case, P(O*_x_*) is shifted to a more optimized NO_2_ position leading to a higher P(O*_x_*) production rate, which is associated with enhanced anthropogenic activities as discussed before. The results demonstrate that the coexisting high NO*_x_* and anthropogenic monoterpene largely enhance ozone production in eastern China.

Very importantly, in this constructed theoretical framework, we can then predict the ozone enhancement ratio for different monoterpene emission scenarios, as well as changing NO*_x_* concentrations, to perform a meta-analysis of available previous field campaigns with high monoterpene concentrations reported. Figure 4 shows that monoterpene oxidation could enhance the P(O*_x_*) by 6% on campaign average in East China, which may increase to 16% if observed sum monoterpenes are identified as limonene (Fig. S7), while the enhancement is usually below 2% in other studies, or even caused ozone net loss for forest environments due to the limited NO*_x_* conditions. A recent study found significant amounts of volatile chemical product emissions [25] for New York City, of which monoterpene was a major component with a daytime maximum of ∼0.08 ppb. However, the influence of monoterpene oxidation on ozone production for the New York case is marginal due to its low NO*_x_* level (Fig. 4, letter H). The conditions for the two megacities (Mexico City and Delhi, denoted as the letters G and F in Fig. 4) of the developing countries are very similar, located on the right side of the NO_2_ dependence curve. The enhancement is 2%–6% for Mexico City and up to 8%–36% for Delhi (see Fig. 4 and Fig. S7). Our generalized theoretical framework highlights the importance of the coexistence of appropriate monoterpenes and NO*_x_* for fast ozone production from the monoterpene oxidations. The high monoterpene accompanied by a moderate or high level of NO*_x_* seems to be a unique feature of developing countries (such as China and India), which may be attributed to intensive and increasing human activities. This implies that anthropogenic monoterpene emissions may play a more significant role in ozone pollution in developing countries. Recently, a wildfire was reported to emit monoterpenes in the USA and influence the atmospheric chemistry [52,53], indicating that natural burning events (like forest and savanna fires) might also result in significant monoterpene emissions and impact the formation of secondary pollutants.

## CONCLUSIONS AND IMPLICATIONS

Our results show that anthropogenic activities, mostly biomass burning, emit large amounts of monoterpenes in eastern China during summer time, which leads to a significant enhancement of ozone production along with an elevated NO*_x_* level. Our study highlights that urban ozone pollution control could be more challenging than expected if anthropogenic monoterpenes are considered. Given that monoterpenes also strongly contribute to secondary aerosol formations [54,55], our findings suggest that the control of anthropogenic monoterpene emissions may provide a critical policy tool for achieving the joint control of ozone and particle pollution. Besides, for future carbon neutrality pathways in China, the coordinated reduction of NO*_x_* and VOCs, including monoterpenes, should also be considered for mitigation of air pollution and climate change. In future works, emission flux observations and speciated monoterpene observations are urgently needed to quantify anthropogenic monoterpenes directly. The measurement of speciated monoterpenes will also be very helpful in diagnosing ozone-NO_x_-VOC sensitivities. Moreover, setting up the speciated anthropogenic monoterpene emission inventory will be critical if the models are to simulate secondary pollution and plan for mitigation strategies regionally.

## MATERIALS AND METHODS

### Monoterpene measurements

A comprehensive suite of trace gas compounds and aerosol properties was measured in the field campaign. The instrument description and details of the sampling site can be found in Text S1. Monoterpene was measured by a commercial Proton Transfer Reaction with Quadruple Interface Time-of-Flight Mass Spectrometer (PTR-Qi-TOF, IONICON Analytik GmbH, Austria). PTR-Qi-TOF was operated in the m/z from 0 to 530, with a mass resolution of 3500–5500 at m/z 45–204. The drift tube was operated at 850 V with a pressure of 3.8 mbar at 80°C. Calibrations for monoterpenes were performed by α-pinene in mixed gas standards (Spectra Gases Inc.) at five concentration levels (1.0, 2.5, 5.0, 7.0 and 10.0 ppbv). The sensitivity of α-pinene was 745.3 ncps ppbv^–1^ resulting in an limit of detection (LOD) of 6 ppt at 10 s resolution. Data were analyzed using Tofwerk software v2.5.7 (Tofwerk AG) for high-resolution peak fitting. Signal intensity was normalized by the signal of H_3_O^+^ ion and water clusters. The mixing ratios of monoterpenes were calculated using the ratio of the normalized signal intensity (unit, ncps) to the sensitivity of α-pinene, assuming all monoterpenes had the same detection sensitivity as α-pinene.

### Numerical chemical model

An observation-constrained box model based on the RACM2 [56], with some modifications, is applied in this study. The isoprene mechanism is replaced according to the latest Leuven-Isoprene-Mechanism [57]. A detailed description of the implementation of RACM2 can be found in a previous publication for the summertime campaign in Wangdu, China [30]. In this study, the model calculations are constrained to measurements of nitrous acid (HONO), NO_2_, NO, O_3_, CO, SO_2_, C_2 _− C_12_ VOC and certain oxygenated VOCs such as HCHO, acetaldehyde, glyoxal and acetone, as well as to measured photolysis frequencies, temperature and pressure, and water vapor concentrations. The CH_4_ and H_2_ mixing ratios are assumed to be 1.9 ppm and 550 ppb, respectively. The measured monoterpenes are identified as α-pinene in the model with monoterpenes constrained if not specified. An additional sensitivity test allocating the monoterpenes to limonene is also performed. The model is operated in a time-dependent mode, in which constrained values are updated every 5 min. For all species that are produced in the model, an additional sink representing physical loss processes like dry deposition is implemented at a rate equivalent to a lifetime of 8 h. Furthermore, heterogeneous loss of dinitrogen pentoxide due to uptake into aerosol particles is also considered.

The photochemical O_3_ production rate, P(O*_x_*), is calculated by the difference between the NO_2_ production rates from the reactions of peroxy radicals with NO (F(O*_x_*), Equation 1) and the O*_x_* loss rates from the reactions of OH with NO_2_, ozone with alkenes and NO_3_ with alkenes, as well as ozone photolysis, etc. (D(O*_x_*), Equation 2). Therein, the rate constant *k*_HO_2_+NO_ is taken from NASA JPL Publication 15–10. The rate constants (*k*_RO_2_i+NO_) and NO_2_ yields (α_i_) for speciated RO_2_ are taken from RACM2. The O*_x_* losses from NO_3_ with alkenes are calculated based on the NO_3_ steady-state assumption, and each NO_3_ destruction is considered to consume two ozone molecules:

(1) \begin{eqnarray*} {\rm{F(}}{{\rm{O}}}_{\rm{x}}) &=& {{{k}}}_{{\rm{H}}{{\rm{O}}}_{\rm{2}}{\rm{ + NO}}}\ \left[ {{\rm{H}}{{\rm{O}}}_{\rm{2}}} \right]\ \left[ {{\rm{NO}}} \right]\nonumber\\ && +\, \mathop \sum \limits_{\rm{i}} {\rm{\ }}{{{k}}}_{{\rm{R}}{{\rm{O}}}_{\rm{2}}{\rm{i + NO}}}{\rm{\ [R}}{{\rm{O}}}_{\rm{2}}{{\rm{]}}}_{\rm{i}}{\rm{\ [NO].}} \end{eqnarray*}

(2) \begin{eqnarray*} \rm{D}({\rm{O}}_{\rm{x}})\ &=& \ J({\rm{O}}^{\rm{1}}\rm{D})\left[ {{{\rm{O}}}_{\rm{3}}} \right] \rm{\ \times \ \varphi}\nonumber\\ && +\, {{{k}}}_{{{\rm{O}}}_{\rm{3}}{\rm{ + Alkenes}}}\ \left[ {{\rm{Alkenes}}} \right]\ \left[ {{{\rm{O}}}_{\rm{3}}} \right]\nonumber\\ && +\, {{{k}}}_{{{\rm{O}}}_{\rm{3}}{\rm{ + OH}}}\ \left[ {{\rm{OH}}} \right]\ \left[ {{{\rm{O}}}_{\rm{3}}} \right]\nonumber\\ && +\, {{{k}}}_{{{\rm{O}}}_{\rm{3}}{\rm{ + H}}{{\rm{O}}}_{\rm{2}}}\ \left[ {{\rm{H}}{{\rm{O}}}_{\rm{2}}} \right]\ \left[ {{{\rm{O}}}_{\rm{3}}} \right]\nonumber\\ && +\, {{{k}}}_{{\rm{OH + N}}{{\rm{O}}}_{\rm{2}}\ }\left[ {{\rm{OH}}} \right]\ \left[ {{\rm{N}}{{\rm{O}}}_{\rm{2}}} \right]\nonumber\\ && +\, 2\ \times \ ({k}_{\rm{NO_2+O_3}}\ \left[ {{\rm{N}}{{\rm{O}}}_{\rm{2}}} \right]\ \left[ {{{\rm{O}}}_{\rm{3}}} \right]\nonumber\\ && -\, {{{k}}}_{{\rm{NO + N}}{{\rm{O}}}_{\rm{3}}}\ \left[ {{\rm{NO}}} \right]\ \left[ {{\rm{N}}{{\rm{O}}}_{\rm{3}}} \right]\nonumber\\ && -\, {{{j}}}_{{\rm{N}}{{\rm{O}}}_{\rm{3}}}[{\rm{N}}{{\rm{O}}}_{\rm{3}}]). \end{eqnarray*}

The dependence of ozone production rate on the NO_2_ concentrations and monoterpene emission rates is calculated using a chemical box model. The chemical mechanisms are the same as described above. To construct a chemical condition representing the eastern China region, the model calculations were constrained to measurements of CO, CH_4_, C_2 _− C_12_ VOC and water vapor concentrations, as well as measured photolysis frequencies, temperature and pressure. The constraints of NO, NO_2_ and oxygenated volatile organic compounds (OVOCs) in the model are removed. HONO concentrations are set proportional to NO_2_ using the mean HONO-to-NO_2_ ratio of 0.086 derived from the measurements. A series of model sensitivity tests with different monoterpene emission rates (identified as either α-pinene or limonene) is performed to extract the P(O*_x_*) dependence on different NO_2_ concentrations (Fig. S8). The direct constraint of monoterpene concentrations in the model to investigate the NO_2_ dependence could lead to flaws in accounting for the interaction between additional monoterpenes input and photochemical activities. Therefore, the model is applied to different monoterpene emission rates, which is more representative of real-world conditions. For each NO_2_ concentration and monoterpene emission rate, a corresponding ozone production rate is derived from the steady-state calculation. For comparison with other campaigns, the P(O*_x_*) for different NO_2_ concentrations and monoterpene emission rates are normalized to the maximum P(O*_x_*) with zero monoterpenes (white circle in Fig. S8). This normalization constructs a general P(O*_x_*) dependence on NO_2_ for different campaigns (Table S2). In the main text, the normalization P(O*_x_*) is shown for comparison between different studies.

## DATA AVAILABILITY

The data underlying this article are available at: https://disk.pku.edu.cn:443/link/C037595A67710 22D79CECBEB86A058F2.

## Supplementary Material

nwac103_Supplemental_File
