# Supplementary material for: Anthropogenic monoterpenes aggravating ozone pollution
Source: Natl Sci Rev. 2022 May 31;9(9):nwac103. doi: 10.1093/nsr/nwac103 (PMC9477203; doi:10.1093/nsr/nwac103)
Supplement: nwac103_Supplemental_File [file nwac103_supplemental_file.docx]

Supplementary Information for

**Anthropogenic monoterpenes aggravating ozone pollution**

Haichao Wang, Xuefei Ma, Zhaofeng Tan, Hongli Wang, Xiaorui Chen, Shiyi Chen, Yaqin Gao, Ying Liu, Yuhan Liu, Xinping Yang, Bin Yuan, Limin Zeng, Cheng Huang, Keding Lu, Yuanhang Zhang

*To whom correspondence should be addressed.

E-mail: [k.lu@pku.edu.cn](mailto:k.lu@pku.edu.cn) (K. L.); [yhzhang@pku.edu.cn](mailto:yhzhang@pku.edu.cn) (Y. Z.)

**This PDF file includes:**

Supporting Text S1

Supporting Figs. S1- S8

Supporting Table. S1- S2

**Text S1**

- 1. **Description of the campaign**

We conducted the field measurement during the EXPLORE-YRD Campaign (EXPeriment on the eLucidation of the atmospheric Oxidation capacity and aerosol foRmation, and their Effects in Yangtze River Delta) in the summer of 2018 in Taizhou (32.56^o^, 119.99^o^), Jiangsu Province. The measurement site is surrounded by fishpond and farmland. The distance between this site and the closest traffic road is more than 0.2 km, and there are no major industrial surroundings. A comprehensive suite of trace gas compounds and aerosol properties was measured, and the related details are listed in table S1. Instruments set up in three containers with the inlets installed on the container roof and about 5 m above the ground.

**1.2 Experimental Setup**

Monoterpene was measured by a commercial proton transfer reaction time of flight mass spectrometer (PTR-Qi-TOF, IONICON Analytik GmbH, Austria) ^1, 2^. A total of 56 VOC was measured using an automated gas chromatograph equipped with mass spectrometry or flame ionization detectors (GC-MS/FID). The spectral actinic photon flux density was measured by a spectroradiometer (SR) ^3^ and used to calculate the photolysis frequencies. NO*_x_* and O_3_ were monitored by commercials instrument (Thermo Electron model 42*i*, 49*i*). PM_2.5_ mass concentration was measured using a standard Tapered Element Oscillating Microbalance (TEOM, 1400A analyzer). Particle number and size distribution (PNSD) was measured by a scanning mobility particle sizer (SMPS, TSI 3936) and an aerosol particle sizer (APS, TSI 3321). SMPS measured the particles in the range between 14 nm and 523 nm in diameter, and APS measured the particles with a diameter range from 597.6 nm to 10.0 $\mu$m. *S_a_* was calculated based on the dry-state particle number and geometric diameter in each size bin (14 nm - 2.5 μm). Wet particle-state *S_a_* for particle hygroscopicity was calculated using a hygroscopic growth factor ^4^. Meteorological data included relevant humidity (RH), temperature, pressure, wind speed, and wind direction were also available. N_2_O_5_ was measured by a cavity-enhanced absorption spectrometer, CEAS ^5^. During the campaign, the air masses influenced by both urban and biogenic emissions and biomass burning. The data presented in this study were collected from May 27^th^ to June 8^th^, 2018. Time is given as Chinese National Standard Time (CNST = UTC + 8 h). Sunrise was at 05:30, and sunset was at 19:00 CNST.

**OH and HO_2_ measurements**. OH and HO_2_ radical concentrations were measured by a laser-induced fluorescence (LIF) instrument that was deployed in previous field measurements successfully ^6-9^. Separate detection cells for OH and HO_2_ were operated at a pressure of 4 *h*Pa. Approximately 20 mW of 308 nm laser radiation generated by a pulsed, frequency-doubled tunable dye laser was sequentially passed through the two cells. Ambient air was sampled into each cell through 0.4 mm nozzles at a flow rate of 1 slpm (slpm = standard litres per minute). In the OH cell, the OH resonance fluorescence induced by the incident 308 nm laser radiation was detected by a multi-channel plate as recently reported OH detection by LIF in a low-pressure cell can potentially suffer from interferences by internally produced OH^10^. Therefore, chemical modulation measurements are suggested to deployed during field campaigns to examine potential interference. We performed such chemical modulation measurement on an ozone polluted day (July 7^th^). No significant OH interference signals were found for the conditions of this campaign. In the HO_2_ cell, HO_2_ was converted to OH by the reaction with added NO and then OH was detected by LIF. When high concentrations of NO are added for complete conversion, part of the atmospheric RO_2_ radicals from long-chain alkanes, alkenes and aromatics can also be converted to OH, thereby producing a significant interference^11^. In the present campaign, this interference was avoided by adding only small concentrations of NO. The HO_2_ cell was operated alternatingly at HO_2_ conversion efficiencies of 10% for 1 minute and 25% for 3 minutes, showing no difference in the HO_2_ concentrations obtained for the two conversion efficiencies. This suggested that interferences from RO_2_ were negligible. The detection limits (signal-to-noise ratio of 2) for 30 s time resolution were 0.6×10^6^ cm^-3^ and 0.1×10^8^ cm^-3^ for OH and HO_2_, respectively. The accuracies of the radical concentrations were calculated from the reproducibility of the calibrated sensitivities and the inherent systematic uncertainties of the calibration method, yielding 2σ accuracies of 20% and 26% for OH and HO_2_, respectively.

**Description of the instrument for N_2_O_5_ measurements**

N_2_O_5_ was measured by thermal decomposition - cavity enhanced absorption spectroscopy (CEAS) ^5^. In the custom-built instrument, N_2_O_5_ is converted to NO_3_ at a temperature of 120 ^o^C and then detected as NO_3_ at its UV-VIS absorption maximum around 662 nm at 80 ^o^C. The particle filter at the beginning of the inlet tube was exchanged once per hour under polluted conditions. The limit of detection (LOD) was estimated to be 2.7 pptv (1$\sigma$) with an uncertainty of 19 % for a time resolution of 60 s.

**NO_3_ concentration estimation.** Field NO_3_ data was not available in this campaign. Here we estimated nighttime NO_3_ (named as NO_3_n,_ 19:00-05:30) by the temperature-dependent thermal equilibrium of NO_3_-NO_2_-N_2_O_5_ by constraining field observed NO_2_, N_2_O_5_, and temperature (Eq. S1). The daytime NO_3_ (05:30-19:00) calculated by this method was not possible because N_2_O_5_ concentration was below or near the detection limit in most of the daytime period, which led to the calculated NO_3_ using Eq. 1 was unreliable high during daytime.

$[\mathrm{NO}_{3\_eq}]=\frac{[N_{2}O_{5}]}{Keq\cdot\left[ \mathrm{NO}_{2} \right]}$ (Eq. S1)

To assess the daytime oxidation of monoterpene by NO_3_ during the daytime, we estimated daytime NO_3_ concentration by assuming a steady-state in NO_3_. As the median lifetime of NO_3_ is less than 5 s during daytime, the steady-state can be reached within a short period due to the fast NO_3_ loss rate ^12^. The daytime mixing ratio of NO_3_ (named as NO_3_d_) is then calculated by constraining the production and their loss terms and be expressed in Eq. S2 ^13^.

$[\mathrm{NO}_{3\_ss}]=\frac{k_{NO2+O3}\cdot[\mathrm{NO}_{2}]\cdot[O_{3}]}{k_{\mathrm{NO}3+NO}\cdot\left[ \mathrm{NO} \right]+j\left( \mathrm{NO}_{3} \right)+k_{\mathrm{NO}3+VOCs}\cdot\left[ \mathrm{VOCs} \right]+kN_{2}O_{5}/(Keq\cdot\left[ \mathrm{NO}_{2} \right])}$ (Eq. S2)

The loss term of NO_3_ includes NO, VOCs, and the equivalent loss from N_2_O_5_ heterogeneous uptake. The loss of NO_3_ by RO_2_ and HO_2_ is not considered due to the small contribution^14, 15^. *K*_eq_ is the temperature-dependent equilibrium constant between NO_2_, NO_3,_ and N_2_O_5_. The loss rate of N_2_O_5_ heterogeneous hydrolysis is calculated by Eq. S3. Without the quantification of N_2_O_5_ uptake coefficient, γ_N2O5_, here we used a parameterization method to calculate γ_N2O5_ referred from ^16^, the parameterized average γ_N2O5_ is 0.028 ± 0.012, and *C* is the mean molecular speed of N_2_O_5_ (m/s).

$kN_{2}O_{5}=\frac{\gamma_{N2O5}\cdot S_{a}\cdot C}{4}$  (Eq. S3)

As the highly varied γ_N2O5_ in ambient condition ^17, 18^, the loss of N_2_O_5_ heterogeneous hydrolysis may bring some uncertainties and propagate to the estimated daytime NO_3_ concentration. Therefore, the NO_3_ concentration was combined by NO_3_d_ and NO_3_n_ as mentioned above to achieve better precision.

**Description of the instrument for VOC measurements**

The online measurements of ambient VOC concentrations (C_2_–C_12_ hydrocarbons) were done by a gas chromatography system coupled with a flame ionization and mass spectrometer detection (GC-MS/FID). Measurements had a time resolution of one hour during the campaign. VOC were pre-concentrated in an ultralow temperature, cryogen-free pre-concentration device. Detailed analytical methods and quality-assurance quality-control (QA–QC) procedures for this system have been described elsewhere. Detection limits for various compounds were in the range of (0.005–0.070) ppb with an uncertainty of measurements was (10-15) %.

**Description of the instruments for aerosol surface area concentration measurements**

Particle number size distributions (PNSD) were measured by a scanning mobility particle sizer (SMPS) (Long-DMA3081 + CPC3775) and a Nano-SMPS (Nano-DMA3085 + UCPC3776). The multiple charge correction, condensation particle counter (CPC) counting efficiency, and particle loss correction were applied to the measurements. An aerodynamic particle sizer (APS, TSI model 3321, TSI Inc., St. Paul, MN, USA) measured the particle-number size distribution between 500 nm and 10 mm (aerodynamic diameter). The APS results were transformed from aerodynamic to Stokes diameters using a particle density of 1.5 g cm^-3^. The dry-state aerosol surface area was calculated based on the dry-state particle number and geometric diameter in each size bin (<2.5$\mu$m) with a time resolution of 5 min.

Dry-state aerosol surface area was calculated based on PNSD, and corrected to ambient (wet) particle aerosol surface area accounting for particle hygroscopic growth. The growth factor was estimated by the ISORROPIA-II aerosol thermodynamics model ^19^. The model input included water-soluble ions, along with simultaneously measured RH and T. The aerosol solutions were assumed to be metastable. Firstly, the model calculated the mass of aerosol liquid water content (ALWC), then determined the growth factor by taking the cube root of the ratio of the wet to the dry aerosol mass. The overall accuracy for the deduced ambient particle aerosol surface area was estimated to be 30%.

**Description of the instruments for determining photolysis frequencies**

Photolysis frequencies were calculated from spectral actinic flux densities measured with a spectroradiometer (Meteorologie Consult). The instrument employed a quartz receiver, a monolithic single monochromator and a photodiode-array. The setup was calibrated with a PTB-traceable irradiance standard before and after the campaign ^20^. The accuracy of the actinic flux measurements was estimated 10% in the UV range at solar zenith angles smaller than 80°, which is the estimated accuracy for J(O^1^D) and J(NO_2_) as well.

**Description of the instruments for measuring meteorological parameters**

Meteorological parameters including wind speed, wind direction, relative humidity (RH), temperature (T) and pressure (P) were measured with a commercial Met One Meteorological Sensors & Systems. Wind speed and wind direction were measured by Met One 014A and 024A, respectively. RH and T were measured by a multi-parameter sensor system (Met One 083E). The air pressure was measured by Met One 092.

**Table S1.** The observed gas and particle parameters during the campaign.

| Species | Detection of limit | Method | Accuracy |
| --- | --- | --- | --- |
| OH | 0.6 ×10^6^ cm^-3^ (2$\sigma$, 30 s) | LIF | ± 20% |
| HO_2_ | 0.10 ×10^8^ cm^-3^ (2$\sigma$, 30 s) | LIF | ± 26% |
| N_2_O_5_ | 2.7 pptv (1$\sigma$, 1 min) | CEAS | ± 19% |
| NO | 0.1 ppb (3$\sigma$, 1 min) | CL | ± 20% |
| NO_2_ | 0.3 ppb (2$\sigma$, 1 min) | CL | ± 20% |
| O_3_ | 0.5 ppb (2$\sigma$, 1 min) | UV photometry | ± 5% |
| PNSD | 14 nm -700 nm (4 min) | SMPS | ± 20% |
| PM_2.5_ | 0.1 $\mu g$/m^3^ (1 min) | TEOM 1400A | ± 5% |
| VOC | 20-300 pptv (60 min) | GC-MS | ±15% |
| Monoterpene | 6 pptv (10 s) | PTR-Qi-TOF | ±3.5% |
| Isoprene | 6 pptv (10 s) | PTR-Qi-TOF | ±4.0% |
| Photolysis frequencies | 5×10^-5^ /s (1 min) | SR | 10% |

**Table S2.** The data used in Figure. 4

| Campaign | Location | period_  start /LST | period_  end  /LST | MT_total  /ppb | NO_2_  /ppb | O_3_  /ppb | jNO_2_  /s^-1^ | Ref. |
| --- | --- | --- | --- | --- | --- | --- | --- | --- |
| SOAS | Centreville | 12:00 | 12:00 | 0.46 | 0.26 | 32.36 | 0.0051 | ^21^ |
| BEARPEX09 | California | 9:00 | 15:00 | 0.18 | 0.2 | 54 | 0.0051 | ^22^ |
| MCMA2006 | Mexico City | 8:40 | 18:40 | 0.14 | 28.4 | 56.2 | 0.0051 | ^23^ |
| OP3-I | Asian rainforest | 11:00 | 12:00 | 0.4 | 0.13 | 12.5 | 0.0051 | ^24^ |
| GABRIEL | Amazonia rainforest | 14:00 | 17:00 | 0.42 | 0.1 | 17 | 0.0051 | ^25^ |
| PROPHET | Northern Michigan | 10:00 | 11:00 | 0.228 | 0.46 | 40.5 | 0.0051 | ^26^ |
| APHH-India | Delhi | 10:00 | 15:00 | 0.345 | 35 | 70 | 0.0051 | ^27^ |
| NY-ICE | NYC | unknown | unknown | 0.08 | 2 | 84 | 0.0051 | ^28^ |
| This work | East China  (05.27--06.08) | 6:00 | 18:00 | 0.406 | 7.24 | 72.7 | 0.0054 |  |
|  | East China  (05.27--05.30) | 6:00 | 18:00 | 0.592 | 8.76 | 62.4 | 0.0042 |  |
|  | East China  (06.02--06.08) | 6:00 | 18:00 | 0.300 | 6.37 | 78.6 | 0.0057 |  |


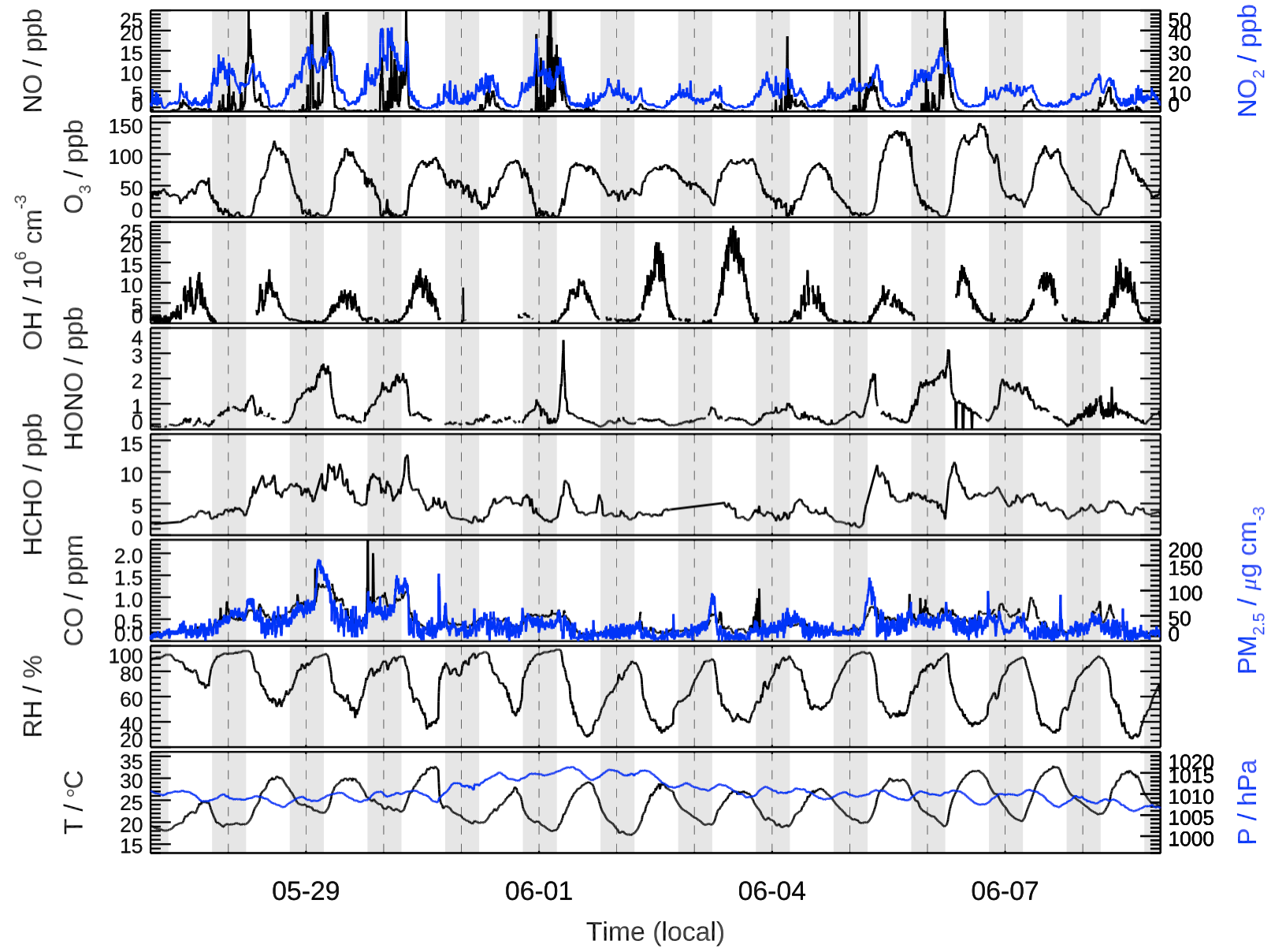


**Fig. S1. Time series of the observed OH radical concentration and typical primary and secondary trace gases.** The panels including NO, NO_2_, O_3_, HONO, HCHO, CO, PM_2.5_, as well as the meteorological parameters, including RH (Relative Humidity), T (Temperature), and P (Pressure). The grey areas denote the nighttime.


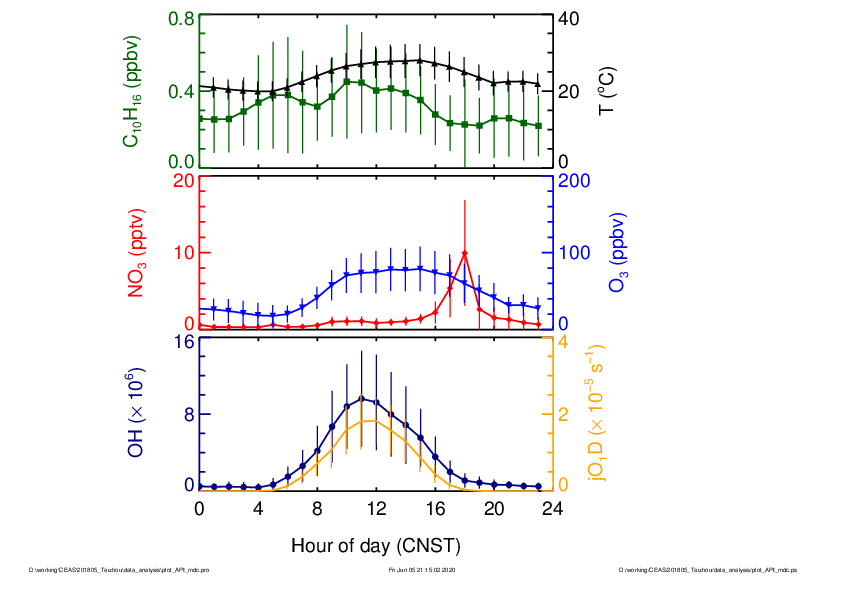


**Fig. S2. Mean diurnal variation of the concentration of NO_3_ (estimated), O_3_, OH, and *j*O^1^D.**


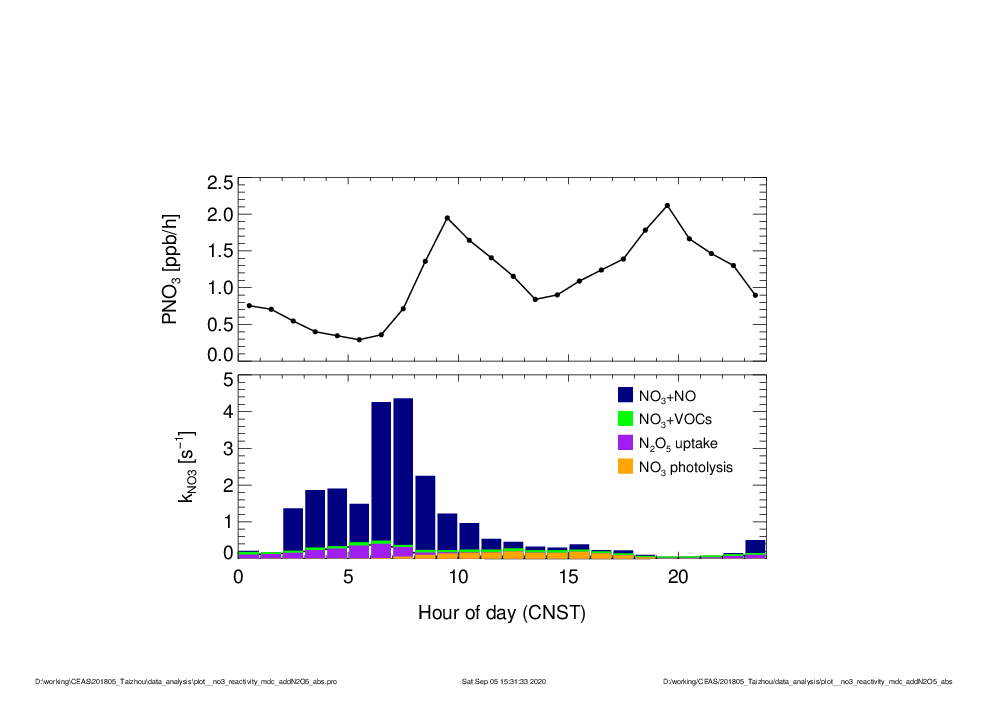


**Fig. S3. The mean diurnal profile of NO_3_ production rate and the loss frequencies of NO_3_ loss terms.** The daytime maximum and averaged NO_3_ production rates are 5.6 ppb/h and 1.2 ppb/h, respectively, while the dominant daytime NO_3_ loss caused by NO is small, with a median loss frequency of 0.16 /s.


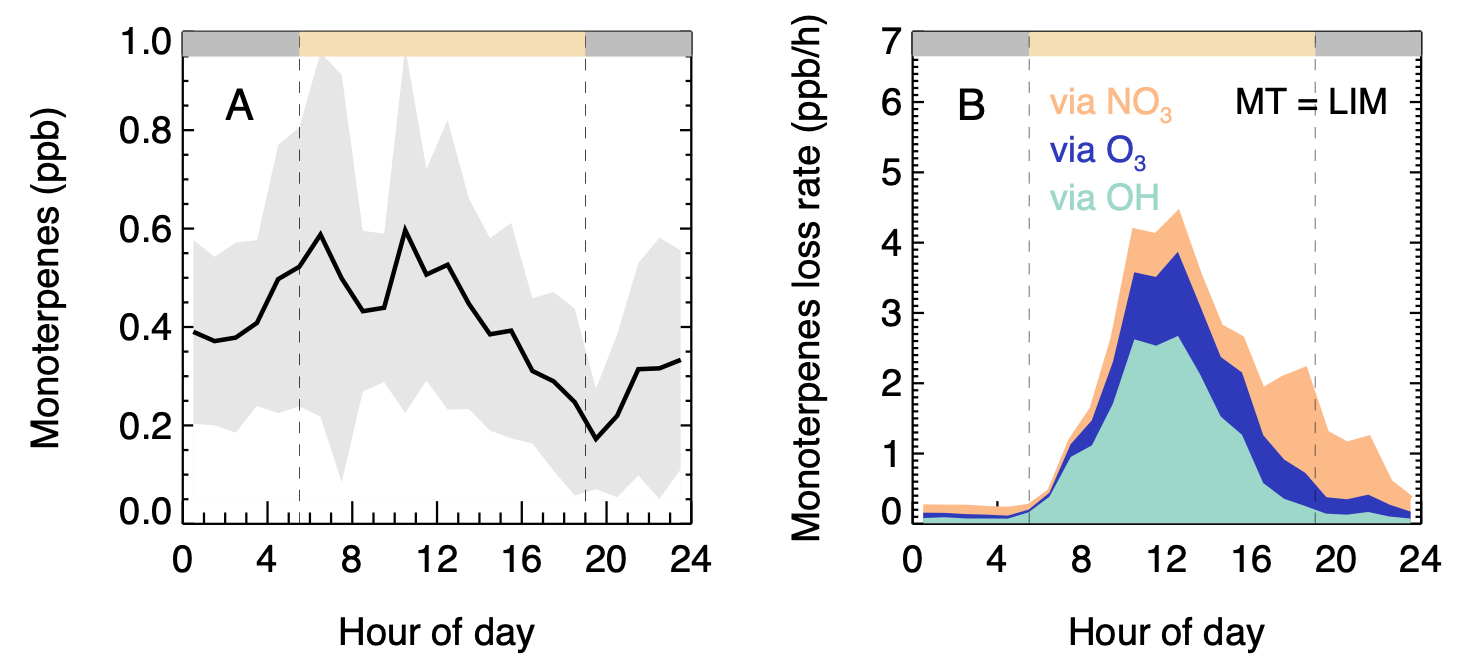


**Fig. S4** Same as Fig. 1B but allocated monoterpenes to be limonene.


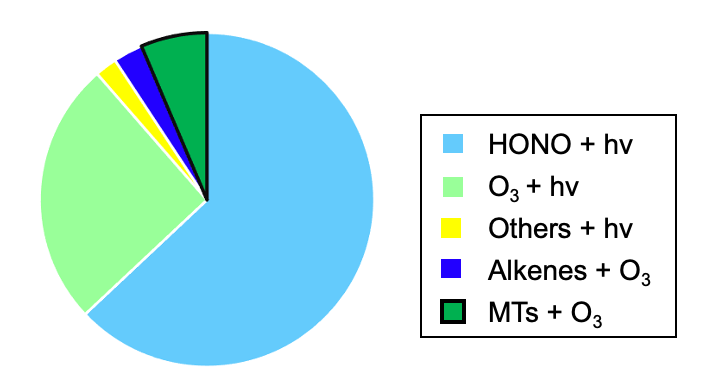


**Fig. S5** The constitutions of OH primary sources during daytime (08:00-18:00). Alkenes+O_3_ represents the ozonolysis of alkenes other than monoterpenes.


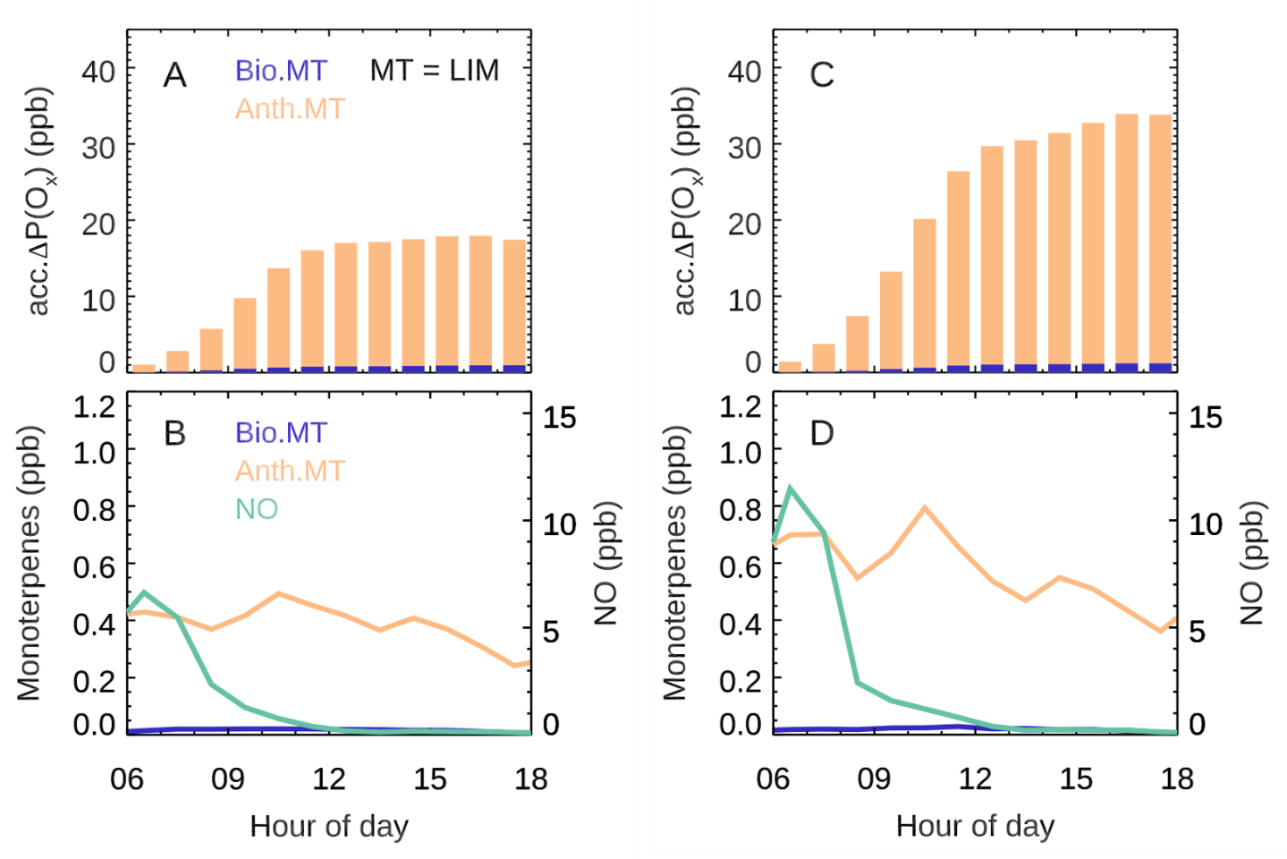


**Fig. S6** Same as Fig. 3 but allocated monoterpenes to be limonene.


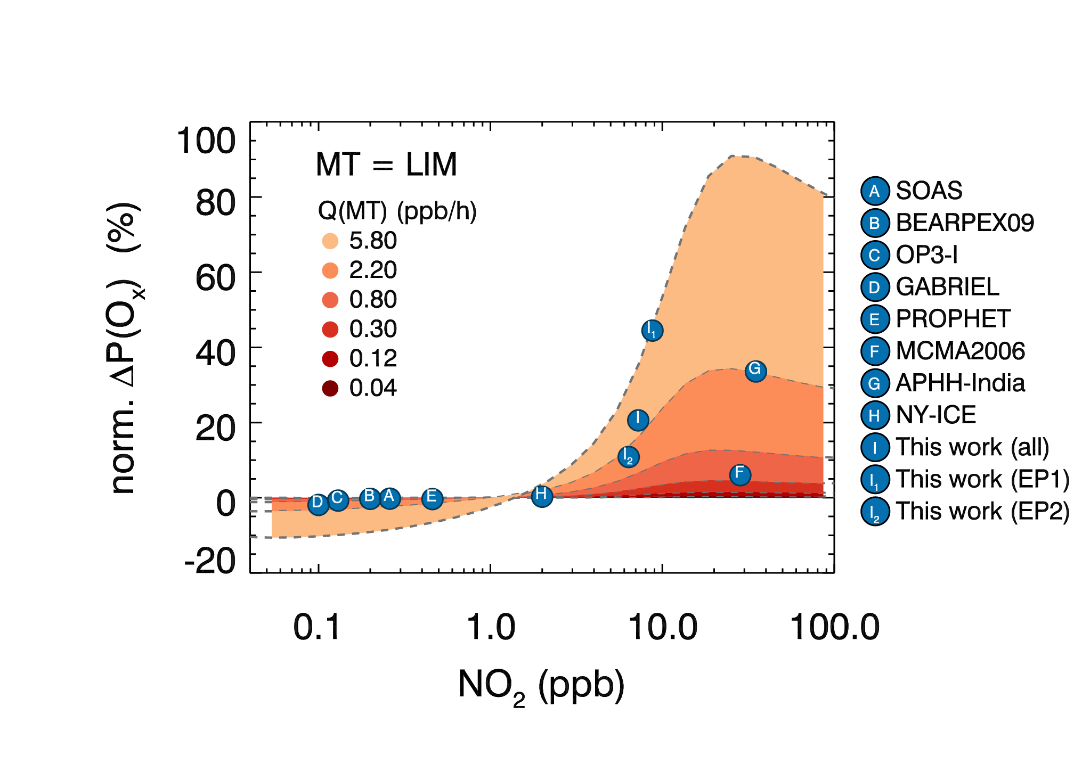


**Fig. S7** Same as Fig. 4 but allocated monoterpenes to be limonene.


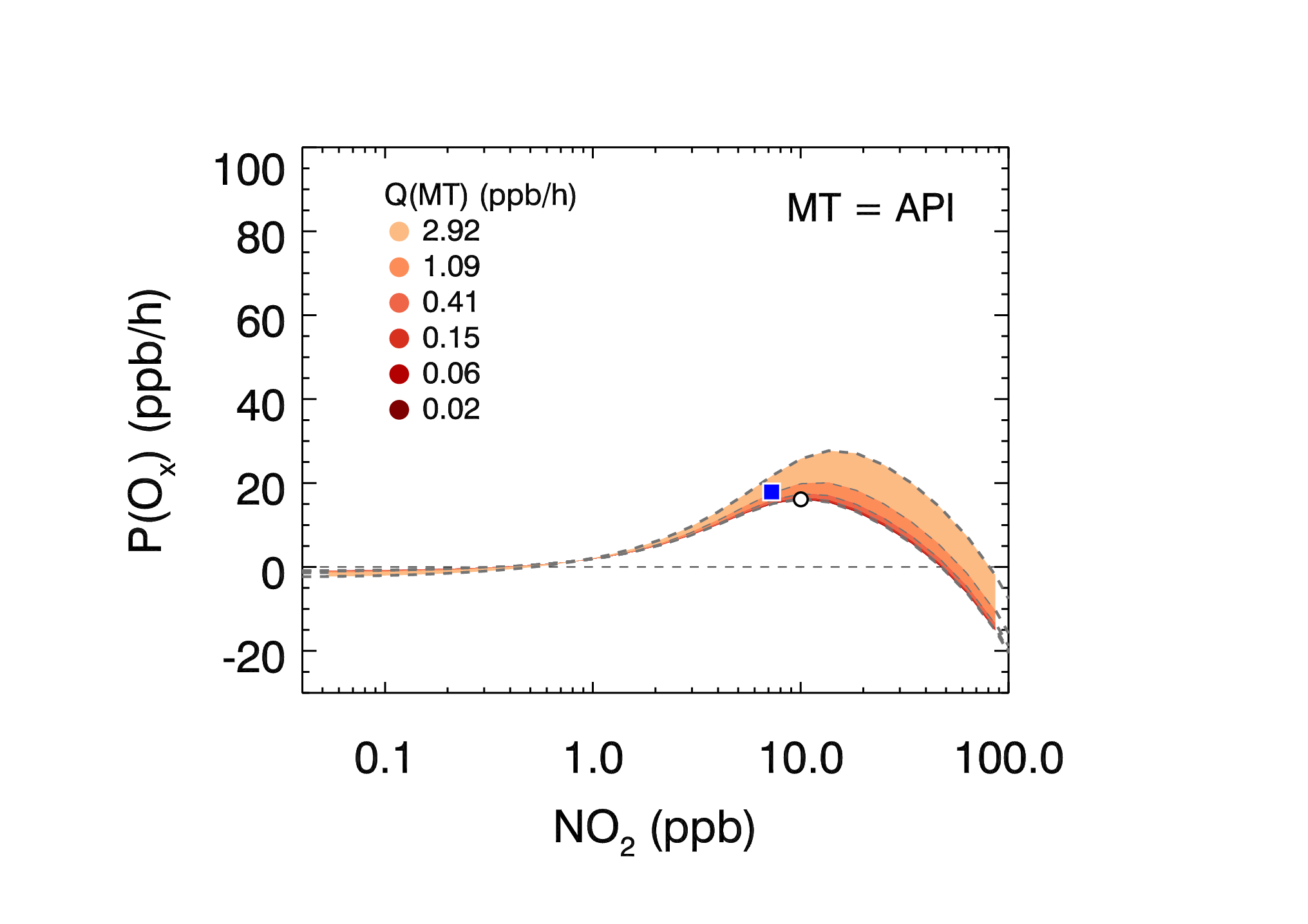

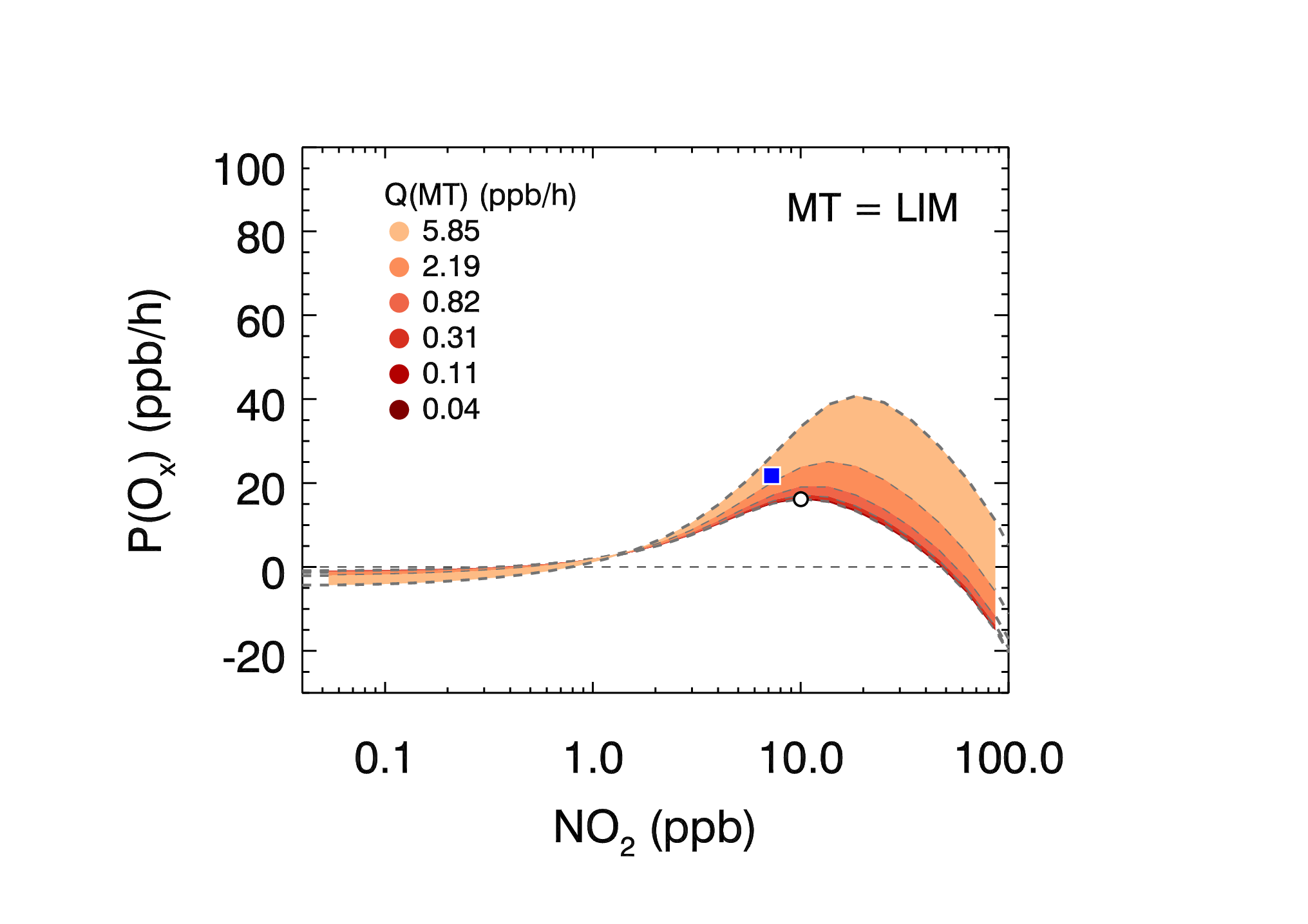


**Fig. S8. The NO_2_ dependence of P(O*_x_*) calculated from the averaged boundary conditions for this campaign.** The white circles denote the maximum P(O*_x_*) with MT=0. The blue square represents the averaged NO_2_ concentrations and monoterpenes emission rates for this campaign. The left and right panel shows the case of α-pinene and limonene, respectively.

**References**

1. Sulzer, P.; Hartungen, E.; Hanel, G.; Feil, S.; Winkler, K.; Mutschlechner, P.; Haidacher, S.; Schottkowsky, R.; Gunsch, D.; Seehauser, H.; Striednig, M.; Jurschik, S.; Breiev, K.; Lanza, M.; Herbig, J.; Mark, L.; Mark, T. D.; Jordan, A., A Proton Transfer Reaction-Quadrupole interface Time-Of-Flight Mass Spectrometer (PTR-QiTOF): High speed due to extreme sensitivity. *Int J Mass Spectrom* **2014,** *368*, 1-5.

2. Yuan, B.; Koss, A. R.; Warneke, C.; Coggon, M.; Sekimoto, K.; de Gouw, J. A., Proton-Transfer-Reaction Mass Spectrometry: Applications in Atmospheric Sciences. *Chem Rev* **2017,** *117*, (21), 13187-13229.

3. Bohn, B.; Corlett, G. K.; Gillmann, M.; Sanghavi, S.; Stange, G.; Tensing, E.; Vrekoussis, M.; Bloss, W. J.; Clapp, L. J.; Kortner, M.; Dorn, H. P.; Monks, P. S.; Platt, U.; Plass-Dulmer, C.; Mihalopoulos, N.; Heard, D. E.; Clemitshaw, K. C.; Meixner, F. X.; Prevot, A. S. H.; Schmitt, R., Photolysis frequency measurement techniques: results of a comparison within the ACCENT project. *Atmospheric Chemistry and Physics* **2008,** *8*, (17), 5373-5391.

4. Liu, X. G.; Gu, J. W.; Li, Y. P.; Cheng, Y. F.; Qu, Y.; Han, T. T.; Wang, J. L.; Tian, H. Z.; Chen, J.; Zhang, Y. H., Increase of aerosol scattering by hygroscopic growth: Observation, modeling, and implications on visibility. *Atmospheric Research* **2013,** *132*, 91-101.

5. Wang, H. C.; Chen, J.; Lu, K. D., Development of a portable cavity-enhanced absorption spectrometer for the measurement of ambient NO3 and N2O5: experimental setup, lab characterizations, and field applications in a polluted urban environment. *Atmos Meas Tech* **2017,** *10*, (4), 1465-1479.

6. Tan, Z.; Fuchs, H.; Lu, K.; Hofzumahaus, A.; Bohn, B.; Broch, S.; Dong, H.; Gomm, S.; Haeseler, R.; He, L.; Holland, F.; Li, X.; Liu, Y.; Lu, S.; Rohrer, F.; Shao, M.; Wang, B.; Wang, M.; Wu, Y.; Zeng, L.; Zhang, Y.; Wahner, A.; Zhang, Y., Radical chemistry at a rural site (Wangdu) in the North China Plain: observation and model calculations of OH, HO2 and RO2 radicals. *Atmospheric Chemistry and Physics* **2017,** *17*, (1), 663-690.

7. Tan, Z.; Lu, K.; Hofzumahaus, A.; Fuchs, H.; Bohn, B.; Holland, F.; Liu, Y.; Rohrer, F.; Shao, M.; Sun, K.; Wu, Y.; Zeng, L.; Zhang, Y.; Zou, Q.; Kiendler-Scharr, A.; Wahner, A.; Zhang, Y., Experimental budgets of OH, HO2, and RO2 radicals and implications for ozone formation in the Pearl River Delta in China 2014. *Atmos Chem Phys* **2019,** *19*, (10), 7129-7150.

8. Tan, Z.; Rohrer, F.; Lu, K.; Ma, X.; Bohn, B.; Broch, S.; Dong, H.; Fuchs, H.; Gkatzelis, G. I.; Hofzumahaus, A.; Holland, F.; Li, X.; Liu, Y.; Liu, Y.; Novelli, A.; Shao, M.; Wang, H.; Wu, Y.; Zeng, L.; Hu, M.; Kiendler-Scharr, A.; Wahner, A.; Zhang, Y., Wintertime photochemistry in Beijing: observations of ROx radical concentrations in the North China Plain during the BEST-ONE campaign. *Atmospheric Chemistry and Physics* **2018,** *18*, (16), 12391-12411.

9. Ma, X.; Tan, Z.; Lu, K.; Yang, X.; Liu, Y.; Li, S.; Li, X.; Chen, S.; Novelli, A.; Cho, C.; Zeng, L.; Wahner, A.; Zhang, Y., Winter photochemistry in Beijing: Observation and model simulation of OH and HO2 radicals at an urban site. *Sci Total Environ* **2019,** *685*, 85-95.

10. Feiner, P. A.; Brune, W. H.; Miller, D. O.; Zhang, L.; Cohen, R. C.; Romer, P. S.; Goldstein, A. H.; Keutsch, F. N.; Skog, K. M.; Wennberg, P. O.; Nguyen, T. B.; Teng, A. P.; DeGouw, J.; Koss, A.; Wild, R. J.; Brown, S. S.; Guenther, A.; Edgerton, E.; Baumann, K.; Fry, J. L., Testing Atmospheric Oxidation in an Alabama Forest. *J Atmos Sci* **2016,** *73*, (12), 4699-4710.

11. Fuchs, H.; Bohn, B.; Hofzumahaus, A.; Holland, F.; Lu, K. D.; Nehr, S.; Rohrer, F.; Wahner, A., Detection of HO2 by laser-induced fluorescence: calibration and interferences from RO2 radicals. *Atmos Meas Tech* **2011,** *4*, (6), 1209-1225.

12. Brown, S. S.; Stark, H.; Ravishankara, A. R., Applicability of the steady state approximation to the interpretation of atmospheric observations of NO3 and N2O5. *Journal of Geophysical Research-Atmospheres* **2003,** *108*, (D17).

13. Osthoff, H. D.; Sommariva, R.; Baynard, T.; Pettersson, A.; Williams, E. J.; Lerner, B. M.; Roberts, J. M.; Stark, H.; Goldan, P. D.; Kuster, W. C.; Bates, T. S.; Coffman, D.; Ravishankara, A. R.; Brown, S. S., Observation of daytime N2O5 in the marine boundary layer during New England Air Quality Study - Intercontinental Transport and Chemical Transformation 2004. *Journal of Geophysical Research-Atmospheres* **2006,** *111*, (D23).

14. Brown, S. S.; Dube, W. P.; Peischl, J.; Ryerson, T. B.; Atlas, E.; Warneke, C.; de Gouw, J. A.; Hekkert, S. T.; Brock, C. A.; Flocke, F.; Trainer, M.; Parrish, D. D.; Feshenfeld, F. C.; Ravishankara, A. R., Budgets for nocturnal VOC oxidation by nitrate radicals aloft during the 2006 Texas Air Quality Study. *Journal of Geophysical Research-Atmospheres* **2011,** *116*.

15. Wang, H.; Lu, K.; Tan, Z.; Sun, K.; Li, X.; Hu, M.; Shao, M.; Zeng, L.; Zhu, T.; Zhang, Y., Model simulation of NO3, N2O5 and ClNO2 at a rural site in Beijing during CAREBeijing-2006. *Atmospheric Research* **2017,** *196*, 97-107.

16. Evans, M. J.; Jacob, D. J., Impact of new laboratory studies of N2O5 hydrolysis on global model budgets of tropospheric nitrogen oxides, ozone, and OH. *Geophysical Research Letters* **2005,** *32*, (9).

17. McDuffie, E. E.; Fibiger, D. L.; Dube, W. P.; Lopez-Hilfiker, F.; Lee, B. H.; Thornton, J. A.; Shah, V.; Jaegle, L.; Guo, H. Y.; Weber, R. J.; Reeves, J. M.; Weinheimer, A. J.; Schroder, J. C.; Campuzano-Jost, P.; Jimenez, J. L.; Dibb, J. E.; Veres, P.; Ebben, C.; Sparks, T. L.; Wooldridge, P. J.; Cohen, R. C.; Hornbrook, R. S.; Apel, E. C.; Campos, T.; Hall, S. R.; Ullmann, K.; Brown, S. S., Heterogeneous N2O5 Uptake During Winter: Aircraft Measurements During the 2015 WINTER Campaign and Critical Evaluation of Current Parameterizations. *Journal of Geophysical Research-Atmospheres* **2018,** *123*, (8), 4345-4372.

18. Tham, Y. J.; Wang, Z.; Li, Q. Y.; Wang, W. H.; Wang, X. F.; Lu, K. D.; Ma, N.; Yan, C.; Kecorius, S.; Wiedensohler, A.; Zhang, Y. H.; Wang, T., Heterogeneous N2O5 uptake coefficient and production yield of ClNO2 in polluted northern China: roles of aerosol water content and chemical composition. *Atmospheric Chemistry and Physics* **2018,** *18*, (17), 13155-13171.

19. Clegg, S. L.; Brimblecombe, P.; Wexler, A. S., Thermodynamic model of the system H+-NH4+-SO42--NO3--H2O at tropospheric temperatures. *J Phys Chem A* **1998,** *102*, (12), 2137-2154.

20. Hofzumahaus, A.; Rohrer, F.; Lu, K. D.; Bohn, B.; Brauers, T.; Chang, C. C.; Fuchs, H.; Holland, F.; Kita, K.; Kondo, Y.; Li, X.; Lou, S. R.; Shao, M.; Zeng, L. M.; Wahner, A.; Zhang, Y. H., Amplified Trace Gas Removal in the Troposphere. *Science* **2009,** *324*, (5935), 1702-1704.

21. Qin, M. M.; Hu, Y. T.; Wang, X. S.; Vasilakos, P.; Boyd, C. M.; Xu, L.; Song, Y.; Ng, N. L.; Nenes, A.; Russell, A. G., Modeling biogenic secondary organic aerosol (BSOA) formation from monoterpene reactions with NO3: A case study of the SOAS campaign using CMAQ. *Atmospheric Environment* **2018,** *184*, 146-155.

22. Mao, J.; Ren, X.; Zhang, L.; Van Duin, D. M.; Cohen, R. C.; Park, J. H.; Goldstein, A. H.; Paulot, F.; Beaver, M. R.; Crounse, J. D.; Wennberg, P. O.; DiGangi, J. P.; Henry, S. B.; Keutsch, F. N.; Park, C.; Schade, G. W.; Wolfe, G. M.; Thornton, J. A.; Brune, W. H., Insights into hydroxyl measurements and atmospheric oxidation in a California forest. *Atmos Chem Phys* **2012,** *12*, (17), 8009-8020.

23. Dusanter, S.; Vimal, D.; Stevens, P. S.; Volkamer, R.; Molina, L. T.; Baker, A.; Meinardi, S.; Blake, D.; Sheehy, P.; Merten, A.; Zhang, R.; Zheng, J.; Fortner, E. C.; Junkermann, W.; Dubey, M.; Rahn, T.; Eichinger, B.; Lewandowski, P.; Prueger, J.; Holder, H., Measurements of OH and HO2 concentrations during the MCMA-2006 field campaign - Part 2: Model comparison and radical budget. *Atmos Chem Phys* **2009,** *9*, (18), 6655-6675.

24. Langford, B.; Misztal, P. K.; Nemitz, E.; Davison, B.; Helfter, C.; Pugh, T. A. M.; MacKenzie, A. R.; Lim, S. F.; Hewitt, C. N., Fluxes and concentrations of volatile organic compounds from a South-East Asian tropical rainforest. *Atmos Chem Phys* **2010,** *10*, (17), 8391-8412.

25. Lelieveld, J.; Butler, T. M.; Crowley, J. N.; Dillon, T. J.; Fischer, H.; Ganzeveld, L.; Harder, H.; Lawrence, M. G.; Martinez, M.; Taraborrelli, D.; Williams, J., Atmospheric oxidation capacity sustained by a tropical forest. *Nature* **2008,** *452*, (7188), 737-740.

26. Tan, D.; Faloona, I.; Simpas, J. B.; Brune, W.; Shepson, P. B.; Couch, T. L.; Sumner, A. L.; Carroll, M. A.; Thornberry, T.; Apel, E.; Riemer, D.; Stockwell, W., HOx budgets in a deciduous forest: Results from the PROPHET summer 1998 campaign. *J Geophys Res-Atmos* **2001,** *106*, (D20), 24407-24427.

27. Nelson, B. S.; Stewart, G. J.; Drysdale, W. S.; Newland, M. J.; Vaughan, A. R.; Dunmore, R. E.; Edwards, P. M.; Lewis, A. C.; Hamilton, J. F.; Acton, W. J. F.; Hewitt, C. N.; Crilley, L. R.; Alam, M. S.; Şahin, Ü. A.; Beddows, D. C. S.; Bloss, W. J.; Slater, E.; Whalley, L. K.; Heard, D. E.; Cash, J. M.; Langford, B.; Nemitz, E.; Sommariva, R.; Cox, S.; Shivani; Gadi, R.; Gurjar, B. R.; Hopkins, J. R.; Rickard, A. R.; Lee, J. D., In situ Ozone Production is highly sensitive to Volatile Organic Compounds in the Indian Megacity of Delhi. *Atmos. Chem. Phys. Discuss.* **2021,** *2021*, 1-36.

28. Coggon, M. M.; Gkatzelis, G. I.; McDonald, B. C.; Gilman, J. B.; Schwantes, R. H.; Abuhassan, N.; Aikin, K. C.; Arend, M. F.; Berkoff, T. A.; Brown, S. S.; Campos, T. L.; Dickerson, R. R.; Gronoff, G.; Hurley, J. F.; Isaacman-VanWertz, G.; Koss, A. R.; Li, M.; McKeen, S. A.; Moshary, F.; Peischl, J.; Pospisilova, V.; Ren, X.; Wilson, A.; Wu, Y.; Trainer, M.; Warneke, C., Volatile chemical product emissions enhance ozone and modulate urban chemistry. *Proceedings of the National Academy of Sciences* **2021,** *118*, (32), e2026653118.
